# Hormone Replacement Therapy and Risk of New-Onset Atrial Fibrillation after Myocardial Infarction - A Nationwide Cohort Study

**DOI:** 10.1371/journal.pone.0051580

**Published:** 2012-12-17

**Authors:** Ditte-Marie Bretler, Peter Riis Hansen, Jesper Lindhardsen, Ole Ahlehoff, Charlotte Andersson, Thomas Bo Jensen, Jakob Raunsø, Christian Torp-Pedersen, Gunnar Hilmar Gislason

**Affiliations:** 1 Department of Cardiology, Copenhagen University Hospital Gentofte, Hellerup, Denmark; 2 Faculty of Health Sciences, Panum Institute, University of Copenhagen, Copenhagen, Denmark; 3 Department of Internal Medicine, Copenhagen University Hospital Holbæk, Holbæk, Denmark; Indiana University School of Medicine, United States of America

## Abstract

**Objectives:**

Our aim was to assess the association between use of hormone replacement therapy (HRT) and risk of new-onset atrial fibrillation (AF) after myocardial infarction.

**Design, Setting and Participants:**

We used Danish nationwide registers of hospitalizations and prescriptions to identify all women admitted with myocardial infarction in the period 1997 to 2009 and with no known diagnosis of AF. Their use of overall HRT and HRT categories was assessed. Multivariable Cox proportional hazards analysis was used to calculate the risk of new-onset AF first year after discharge, comparing use of HRT to no use.

**Main Outcome Measures:**

New-onset atrial fibrillation.

**Results:**

In the period 1997 to 2009, 32 925 women were discharged alive after MI. In the first year after MI, new-onset AF was diagnosed in 1381 women (4.2%). Unadjusted incidence rates of AF decreased with use of HRT (incidence rate 37.4 for use of overall HRT and 53.7 for no use). Overall HRT was associated with a decreased risk of AF (HR 0.82, 95% confidence interval [CI] 0.68–1.00). The lowest risk of AF was found in women ≥80 years old for use of overall HRT and vaginal estrogen (HR 0.63, CI 0.42–0.94, and HR 0.58, CI 0.34–0.99, respectively). Decreased risk of AF with use of overall HRT and HRT categories was also found in other age groups.

**Conclusions:**

Use of HRT is associated with a decreased risk of new-onset AF in women with myocardial infarction first year after discharge. The underlying mechanisms remain to be determined. Unmeasured confounding might be one of them.

## Introduction

Atrial fibrillation (AF) is the most common cardiac arrhythmia and the incidence of AF increases with age. [1], [2] Several studies have shown differences between men and women in the natural history of AF, but the gender differences are largely unexplained. [3], [4] AF is associated with increased mortality and morbidity, especially among patients with ischemic heart disease, and exerts a heavy burden on the healthcare system not least due to complications such as heart failure and ischemic stroke. [5] In the setting of acute myocardial infarction (MI), new-onset AF is frequent and carries an approximately 40% excess risk of death, even after adjustment for known risk factors. [6] Hormone replacement therapy (HRT) is widely used to treat menopausal symptoms even though use has dropped markedly after the Heart and Estrogen/progestin Replacement Study trial did not find the expected benefit regarding cardiovascular endpoints and the Women’s Health Initiative (WHI) study reported an increased risk of myocardial infarction, stroke, deep venous thrombosis and breast cancer with use of HRT in 2002. [7], [8], [9], [10] In spite of this general decrease in use of HRT, we have previously found that the percentage of women using HRT at time of MI did not change significantly after 2002. [11] Estrogen might have favorable effects on infarct size and remodeling and an association between HRT and new-onset AF after MI is therefore plausible and would have major public health implications because AF is associated with severe complications and due to the wide use of HRT. [12], [13], [14], [15], [16], [17], [18].

The aim of the present study was therefore to assess whether in a high-risk population of women with MI there was an impact of HRT use on risk of developing AF. We believe our study is the first to explore this hypothesis.

## Methods

### Ethics

Retrospective register studies do not require ethical approval in Denmark. All available data were encrypted to ensure full anonymity and the Danish Data Protection Agency approved the study (No. 2007-41-1667) and waived the need for written informed consent.

### Data Sources

In Denmark, all citizens are provided with a unique and personal civil registration number that enables linkage of national registers on an individual level. The National Patient Register holds information on all admissions to Danish hospitals since 1978 registered by diagnoses according to the World Health Organization’s International Classification of Diseases (ICD). Information of all prescriptions dispensed by Danish pharmacies since 1995 is found in the Danish National Prescription Register and the prescriptions are coded according to the Anatomic Therapeutical Chemical (ATC) system. As all residents in Denmark are covered by a national health security system and have the cost of drugs partially reimbursed, the pharmacies are required to register all dispensed prescriptions. [19] All deaths are registered in the Central Population Register. Operations are classified according to the Danish Classification of Operation until 1996 and from 1997 according to the Nordic Medico-Statistical Committee Classification of Surgical Procedures. [20].

### Population

**Figure 1:**
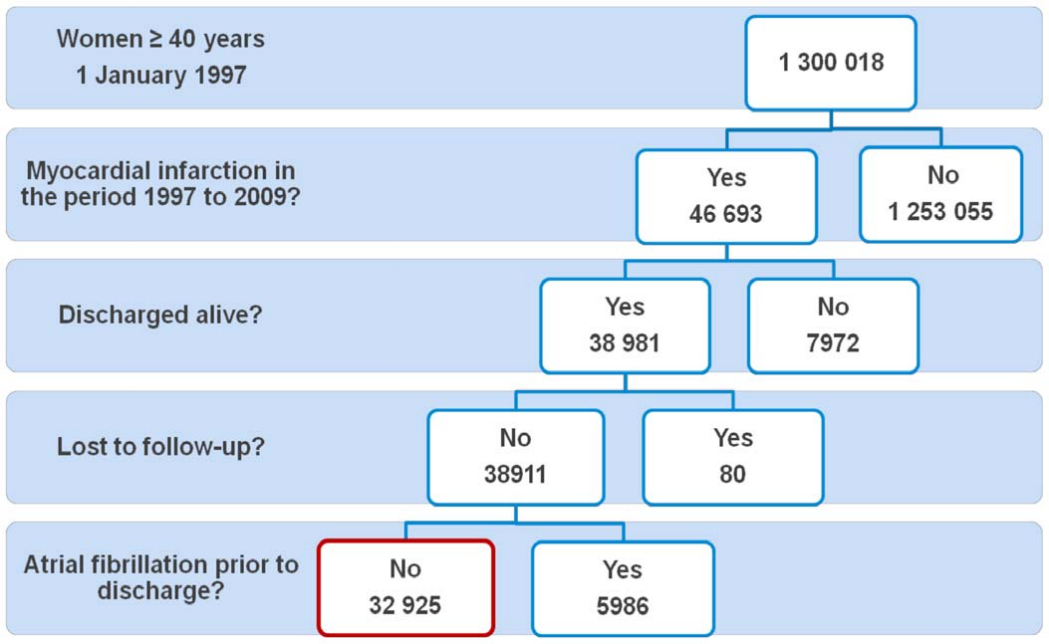
Study population. Red box indicates the final study population.

We identified all women who were 40 years or older on 1 January 1997 and who were hospitalized with a diagnosis of MI (ICD-8 code 410, ICD-10 codes I21 to I22) in the National Patient Register in the period 1997 to 2009. The MI diagnosis has previously been validated and has a sensitivity of 91% and a positive predictive value of 93%. [21] Included in the study were all women who were discharged alive and with no known diagnosis of AF (ICD-8 code 42793 and 42794, ICD-10 code I48) prior to discharge (Figure 1). The yearly household income was calculated as an average of the income during the 5 years prior to admission and divided into income tertiles. We excluded women who migrated to or from Denmark in the study period 1997 to 2009.

### Use of Hormone Replacement Therapy

In the National Prescription Register we identified all claimed prescriptions for HRT (ATC codes G03C, G03D, G03F, G03XC and G02BA03) in the study period. We excluded drugs usually used for contraceptive purposes except for a progesterone intrauterine device (IUD) which is commonly used by postmenopausal women. HRT is a broad spectrum of chemical compounds, formulations, routes of administration and dosages. We divided this pharmacological multiplicity into 4 categories (see Table 1 for details): 1. Estrogen alone; 2. Combinations of estrogen and progestogen; 3. Vaginal estrogen alone; 4. ‘Other HRT’. These categories were mutually exclusive. The National Prescription Register includes information about dispensing date, strength, and quantity of the prescription, but indication and daily drug dose is not included. Estimations of the daily drug doses were therefore made by calculating the average drug dose of up to 3 consecutive prescriptions prior to the actual prescription and not using information of future prescriptions in the calculations. This method has previously been described in detail. [22] On the basis of these assumptions, we calculated periods where HRT was available and we defined patients as receiving HRT if it was available. This means that every woman could contribute with HRT treatment periods several times and that the category of HRT could change over time.

**Table 1:** Categories of hormone replacement therapy.

HRT category	ATC codes
**1. Systemic estrogen**	G03C A03, G03C A04, G03C A53, G03C A57
(Oral, intramuscular, nasal, or transdermal administration)	
**2. Systemic estrogen and progestogen, continuous or cyclic** *	Continuous: G03F A01, G03F A11, G03F A12, G03F A15, G0F A17
(Estrogen: oral, intramuscular, nasal, or transdermal administration. Progestogen: Intrauterine, transdermal, or oral administration)	Cyclic: G03F B01, G03F B05, G03F B06, G03F B09, G03F B11, G03H B01
	Or estrogen from category 1+ progestogen from category 4
**3. Vaginal estrogen**	Vaginal tablet: G03C A03, G03C A04, G03C A57, G03C B01
	Vaginal ring: G03C A03
	Vaginal cream: G03C A04
**4. Other HRT**	Progestogen intrauterine device: G02B A03
	Raloxifene: G03X C01
	Tibolone: G03D C05, G03C X01
	Progestogen alone: G03D A02, G03D A04, G03D B01, G03D C02, G03D C03
	Or any combination of the categories 1 and 3 or 2 and 3

Abbreviation: HRT, hormone replacement therapy; ATC, Anatomical Therapeutic Chemical system.

* Continuous combined estrogen/progestogen: daily doses of both estrogen and progestogen. Cyclic combined estrogen/progestogen: daily doses of estrogen and intermittent periods with daily doses of progestogen.

### Concomitant Pharmacotherapy and Comorbidity

Concomitant pharmacotherapy at baseline was defined as claimed prescriptions within 180 days prior to the MI for anti-thyroid medication (H03B), statins (ATC code C10AA), and clopidogrel (ATC code B01AC04). Comorbidity at baseline was defined using the modified Ontario Acute Myocardial Infarction Prediction Rules by diagnosis from the index admission and 1 year prior to admission. [23], [24] The National Patient Register has a low sensitivity for the heart failure diagnosis and holds no information about left ventricular ejection fraction. [25] Prescriptions for loop diuretics (ATC code C03C) were therefore used as a proxy for heart failure, as previously done. [26] Similarly, we used prescriptions for glucose-lowering medication (ATC A10) as a proxy for diabetes requiring hypoglycemic treatment. We identified women with hypertension at baseline from combination treatment with at least two of the following classes of antihypertensive drugs: α adrenergic blockers (ATC codes C02A, C02B, C02C), non-loop diuretics (ATC codes C02DA, C02L, C03A, C03B, C03D, C03E, C03X, C07C, C07D, C08G, C09BA, C09DA, C09XA52), vasodilators (ATC codes C02DB, C02DD, C02DG, C04, C05), β blockers (ATC code C07), calcium channel blockers (C07F, C08, C09BB, C09DB), and renin-angiotensin system inhibitors (C09). This definition has previously been used and is validated in a Danish population with a positive predictive value of 80.0% and a specificity of 94.7%. [27], [28] Information about valvular disease and operations (ICD-8 codes 394, 395, 396, ICD-10 codes I05, I34, I359, operation codes 4240, 4242, KFK, KFM, and KFP), revascularization (percutaneous coronary intervention [operation codes 30350, 30354 and KFNG], and coronary artery bypass graft surgery [operation codes 30009 to 30199 and KFNA to KFNE]) was also obtained.

### Outcome Measures

New-onset AF was defined as a diagnosis with the ICD-10 code I48 in the period after discharge for myocardial infarction with no prior known diagnosis of AF from 1978 and onwards. Follow-up was one year after discharge or until date of first AF diagnosis, death, or 31 December 2009. The AF diagnosis in The National Patient Register has previously been found to have a sensitivity of 88% and a specificity of 88 to 99%. [29], [30], [31].

### Statistical Analysis

Baseline characteristics are presented as numbers with percentages or medians with interquartile ranges. Age was categorized as the age groups 40 to 59 years, 60 to 79 years and ≥80 years (Table 2)**.**

**Table 2:** Baseline characteristics of study population.

Number of women	32 925 (100)
Median age, years (IQR)	75 (66–82)
**Age group**	
40–59	4309 (13.1)
60–79	17 435 (53.0)
80-	11 181 (34.0)
**Year of MI, n (%)**	
1997–2002	16 181 (49.1)
2003–2009	16 744 (50.9)
**Co-morbidity, n (%)**	
Revascularization before discharge	8235 (25.0)
Hypertension*	10242 (31.1)
Prior valvular disease or valve operation	2301 (7.0)
Cerebrovascular disease **	993 (3.0)
Congestive heart failure **	1037 (3.1)
Malignancy **	683 (2.1)
Cardiac dysrhythmias **	227 (0.7)
Chronic renal failure **	238 (0.7)
Acute renal failure **	126 (0.4)
Diabetes with complications **	392 (1.2)
Pulmonary edema **	107 (0.3)
Shock **	138 (0.4)
**Concomitant pharmacotherapy, n (%)** †	
Statins	4447 (13.5)
Loop diuretics	6987 (21.2)
Clopidogrel	394 (1.2)
Glucose-lowering medication	3850 (11.7)
Anti-thyroid medication	977 (3.0)
**Income group, n (%)** ‡	
1	10 145 (30.8)
2	10 722 (32.6)
3	12 058 (36.6)

* Defined as combined treatment with at least two of the following classes of antihypertensive drugs: α adrenergic blockers, non-loop diuretics, vasodilators, β blockers, calcium channel blockers, and renin-angiotensin system inhibitors.

** According to the Ontario Acute Myocardial Infarction Prediction Rules by diagnosis from the index MI admission and 1 year prior to admission.

† Claimed prescriptions within 180 days prior to the index MI admission.

‡ Income tertiles calculated for all women with MI in the period 1997 to 2009.

Unadjusted incidence rates for new-onset AF were calculated. A multivariable Cox proportional hazards model was used to calculate the hazard ratios (HR) of new-onset AF for both overall HRT and the four HRT categories, using no treatment as reference. The multivariable Cox model was adjusted for time-dependent variables of age, revascularization, valvular disease, statins, loop diuretics, anti-thyroid medication, clopidogrel, and glucose-lowering medication, and for fixed variables of income group, hypertension, and year of MI. The model assumptions for the Cox analysis (i.e. lack of interactions and a valid proportional hazards assumption) were tested and found to be valid unless otherwise stated. Tests for interactions were made for the various HRT groups and age, year of MI, revascularization, valvular disease, and concomitant pharmacotherapy and for year of MI and age. To test the robustness of our results using the short follow-up time of one year we made additional analyses including five years of follow-up.

For all statistical analyses a level of 5% was considered statistically significant. All analyses were performed using SAS statistical software, version 9.2 (SAS Institute Inc., Cary, NC, USA) and Stata version 11 (StataCorp, College Station, TX, USA).

## Results

### Baseline Characteristics

A total of 46 963 women experienced a myocardial infarction in the period 1997 to 2009. Of these, 32 925 were discharged alive, were not lost to follow-up and had no prior AF diagnosis and thus constituted the population of this study (Figure 1). Baseline characteristics are shown in Table 2. In this group of women with a median age of 75 years (interquartile range 66 to 82 years), 5188 (15.8%) used HRT in the first year after MI. Systemic estrogen was used by 1295 (3.9%) women, 1401 (4.3%) used systemic estrogen and progestogen, 2442 (7.4%) used vaginal estrogen and 494 (1.5%) used ‘other HRT’. New-onset AF was diagnosed in 1381 women (4.2%) the first year after MI, 108 of these AF diagnoses were in women using HRT. The women who were diagnosed with AF first year after discharge had a median age of 78 years, and in the age groups 40 to 59 years, 60 to 79 years and ≥80 years there were 47 (1.1%), 758 (4.3%) and 576 (5.2%) women with AF, respectively.

### Overall Risk of Atrial Fibrillation

**Figure 2:**
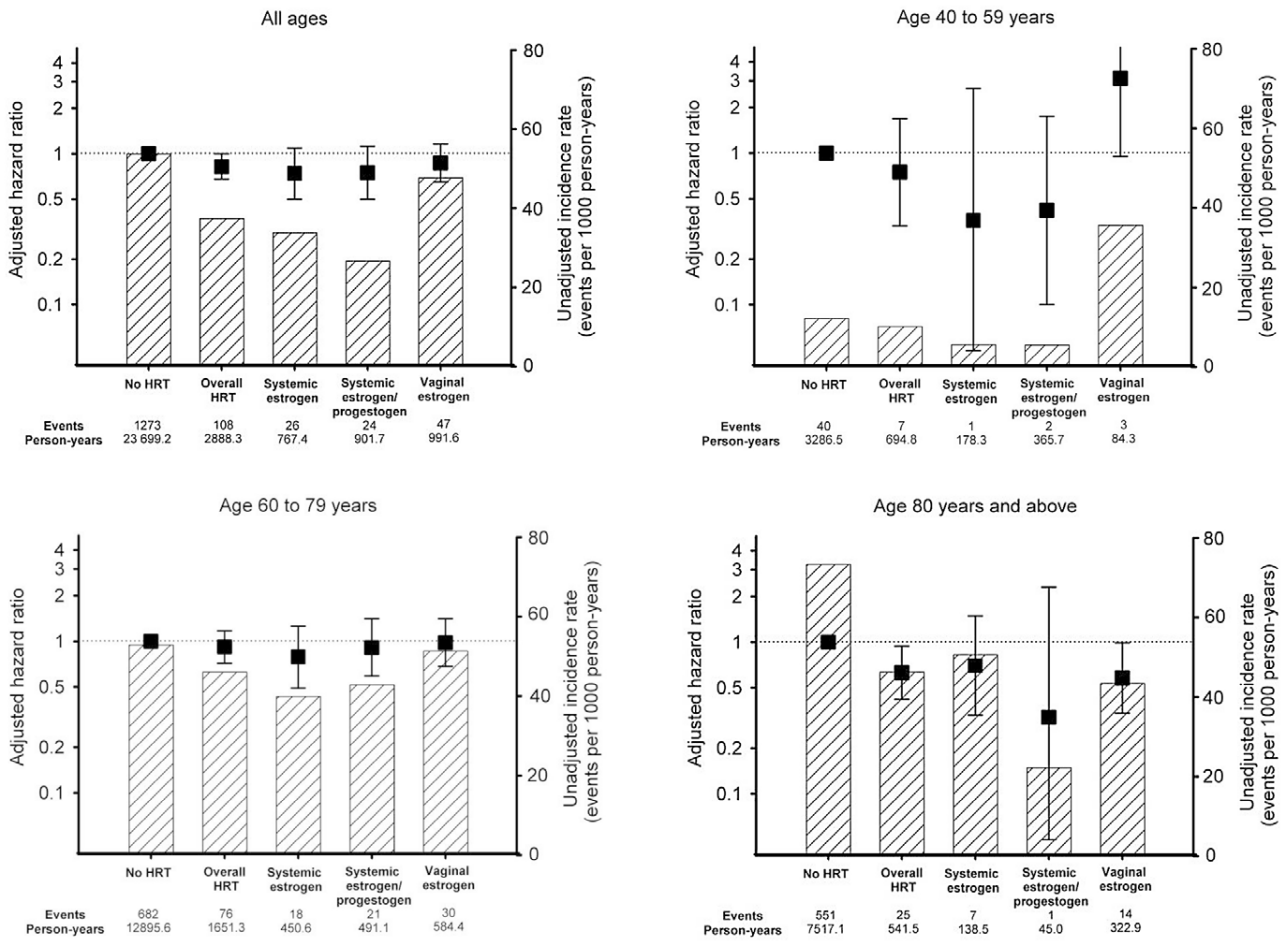
Hazard ratios and unadjusted incidence rates of atrial fibrillation. Black dots with whiskers: Hazard ratio with 95% confidence interval. Hatched columns: Unadjusted incidence rates. Abbreviation: HRT, hormone replacement therapy. Hazard ratios were calculated using a multivariable Cox proportional hazards model adjusted for fixed variables of year of myocardial infarction, hypertension, and income group and time-dependent variables of revascularization, valvular disease, statins, loop diuretics, clopidogrel, anti-thyroid and glucose-lowering medication.

Non-users of HRT had higher unadjusted incidence rates of AF than users of HRT and the rates increased with higher age group in both users and non-users of HRT (Figure 2). Differences were seen between the categories of HRT. We found an interaction between age and HRT use (*p* for interaction<0.001) and the Cox proportional hazards analyses were therefore stratified on age groups. The lowest risk of AF was found in women ≥80 years old for use of overall HRT and vaginal estrogen (HR 0.63, CI 0.42–0.94, and HR 0.58, CI 0.34–0.99, respectively). In the age group 60 to 79 years, use of systemic estrogen, use of systemic estrogen and progestogen and use of vaginal estrogen were associated with a lower risk of AF, but these estimates did not reach statistical significance. The same lower but statistically insignificant risk was found in women ≥80 years old with use of systemic estrogen.

### Other Analysis

When analyzing five years of follow-up, we found the same pattern of higher unadjusted incidence rates with age and with no use of HRT and the multivariable Cox proportional hazards analysis yielded qualitatively similar results as the analysis with one year of follow-up. The risk of AF in women ≥80 years old for use of overall HRT and vaginal estrogen (HR 0.71, CI 0.55 to 0.92, and HR 0.60, CI 0.43 to 0.85, respectively) was still decreased. At baseline, acetyl salicylic acid, vitamin K antagonists, and dipyridamole were used by 21.1%, 1.3% and 2.8% of the women. Inclusion of these variables and of duration of HRT use in the Cox models did not influence the results and these variables where therefore not included in the final Cox models.

## Discussion

The present study is to our knowledge the first to address whether women who use HRT after MI have an altered risk of new-onset AF. Our main finding was that use of overall HRT was associated with a nine to 37 percent decrease in risk of AF the first year after MI, depending on age group. The lowest risks were found in women ≥80 years old. This is important since this age group constitutes a high-risk population, as shown by the incidence rates of AF increasing with higher age group and because AF after MI is frequent and carries an approximately 40% excess risk of death, even after adjustment for known risk factors. [6] In the age group 40 to 59 years there were only 47 cases of new-onset AF the first year after MI. That this age group has fewer events than the other age groups was expected since it has previously been established that the incidence of AF approximately doubles for every 10-year increment in age. [32] However, the few events in this age group make the interpretation of the results of the Cox analyses difficult because the confidence intervals are very wide.

We have not found other reports about an association between HRT and AF, which we find interesting since studies of associations with AF are numerous as are studies of associations between HRT and various diseases. [33], [34], [35], [36], [37], [38], [39], [40], [41] A recent paper suggests that HRT’s potential effects on AF risk needs to be explored. [42] The lacking results either suggest publication bias or a true lack of information.

There are several possible mechanisms through which HRT may modulate the risk of AF after MI. Firstly, estrogen has divergent effects on serum markers of inflammation and there is mounting evidence to support the influence of inflammation on the pathogenesis of AF. [43], [44], [45] Secondly, estrogen might have influence on MI size and post-MI cardiac remodeling through effects on ischemic preconditioning, cardiomyocyte apoptosis, and neovascularization. [12], [13], [14], [15], [16], [17], [18] Thirdly, endothelial dysfunction is associated with AF and may be ameliorated by estrogen. [46], [47], [48] Along this line, we find it possible that our observed decreased risk of AF in post-MI women using HRT might in fact be due to the effects of HRT. However, as this was an observational study we cannot postulate causality of our findings. Also, these results are from a post-MI population with a median age of 75 years and cannot be extrapolated to the general population. The results may be due to unmeasured confounding, e.g., a ‘healthy user effect’ even though a previous Danish study did not find evidence of such effect. [49] The differences in association between atrial fibrillation and the various categories of HRT can either reflect biological differences between HRT categories or that use of some categories of HRT are more associated with a “healthy user effect” than others. The apparent protective effect of vaginal estrogen in the group of women ≥80 years old could be explained as a ‘healthy user effect’ if use of vaginal estrogen is an indicator of, e.g., sexual activity and, by inference, a better general health. Our finding of a possible decreased risk of new-onset atrial fibrillation with use of HRT especially among the elderly needs confirmation in other studies. Bearing in mind that the WHI found hazardous effects of HRT on risk of myocardial infarction, stroke, breast cancer, and deep venous thrombosis, a possible beneficial effect of HRT on risk of new-onset atrial fibrillation after MI may be outweighed and there is currently no indication for using HRT as a protection against new-onset atrial fibrillation after MI. [9].

### Strengths and Limitations

The main strength of this study is the complete and nationwide cohort of all women with MI in the period 1997 to 2009, including information about their HRT use, concomitant pharmacotherapy and comorbidity. The required registration by the Danish pharmacies and the reimbursement of medical expenses ensure that all social classes and women both in and out of the labor market were represented. The conducted sensitivity analyses where follow-up was the first five years after MI yielded similar results. However, there are several limitations to this study inherent to its observational nature. Our calculations of HRT doses and treatment durations can be merely approximations and the true dates of beginning and discontinuing a treatment may differ from the calculated dates. We lack information about the precise indications for HRT and about symptom characteristics which may be a confounder, since hot flashes may be a marker of increased cardiovascular risk. [50] Another limitation is that we have no information regarding clinical parameters such as obesity, menopausal status, smoking status or left ventricular ejection fraction in the registers and although the incorporation of, e.g., concomitant medication (including use of loop diuretics as a proxy for heart failure and use of at least two classes of antihypertensive drugs as a proxy for hypertension), comorbidity and income in our analyses might have captured some effects of these important cardiovascular risk factors, we can not rule out unmeasured confounding. As the Danish population is mainly white we cannot conclude anything about racial or ethnic differences. Finally, our study was limited by lack of power. However, this is the first study to focus on a population of women after MI and the impact of their use of HRT on the risk of new-onset AF, and it seems unlikely that randomized trials or observational studies with a larger population will be conducted.

### Conclusion

Use of HRT first year after MI was associated with a decreased risk of new-onset AF especially among women ≥80 years old. Differences were found between different categories of HRT. The underlying mechanisms remain to be determined, but the decreased risk may be due to protective effects of HRT, a healthy user effect among users of HRT and/or other unmeasured confounders. Our results need confirmation preferably in a randomized clinical trial and there is no indication for using HRT as a protection against new-onset AF after MI.
